# 2-Hy­droxy-*N*′-(4-hy­droxy-3-nitro­benzyl­idene)benzohydrazide

**DOI:** 10.1107/S1600536811000201

**Published:** 2011-01-08

**Authors:** Zhen Zhang

**Affiliations:** a Experimental Center, Linyi University, Linyi Shandong 276005, People’s Republic of China

## Abstract

The title compound, C_14_H_11_N_3_O_5_, crystallized with two independent mol­ecules per asymmetric unit. Each mol­ecule assumes an *E* configuration with respect to the methyl­idene unit. Intra­molecular O—H⋯O and N—H⋯O hydrogen bonds are present in each mol­ecule and they are linked by an O—H⋯O hydrogen bond. The dihedral angles between the mean planes of the two benzene rings are 4.45 (16) and 1.7 (2)° in the two mol­ecules. The crystal structure is stabilized by inter­molecular O—H⋯O and N—H⋯O hydrogen bonds.

## Related literature

For the biological applications of hydrazone compounds, see: Ajani *et al.* (2010 ▶); Avaji *et al.* (2009 ▶); Fan *et al.* (2010 ▶); Rasras *et al.* (2010 ▶). For similar hydrazone compounds, see: Ahmad *et al.* (2010 ▶); Ban (2010 ▶); Ji & Lu (2010 ▶); Shalash *et al.* (2010 ▶).
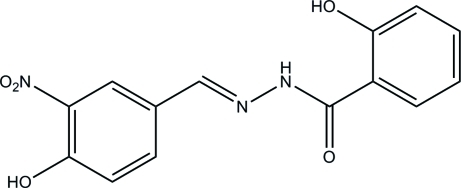


## Experimental

### 

#### Crystal data

C_14_H_11_N_3_O_5_
*M* *_r_* = 301.26
Monoclinic, 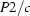

*a* = 13.769 (2) Å
*b* = 13.089 (2) Å
*c* = 19.999 (3) Å
β = 131.426 (3)°
*V* = 2702.5 (7) Å^3^
*Z* = 8
Mo *K*α radiation
μ = 0.12 mm^−1^
*T* = 298 K
0.23 × 0.21 × 0.20 mm

#### Data collection

Bruker SMART APEX CCD area-detector diffractometer
Absorption correction: multi-scan (*SADABS*; Bruker, 2009 ▶) *T*_min_ = 0.974, *T*_max_ = 0.977
20705 measured reflections
5704 independent reflections
3336 reflections with *I* > 2σ(*I*)
*R*_int_ = 0.063

#### Refinement

*R*[*F* ^2^ > 2σ(*F* ^2^)] = 0.074
*wR*(*F* ^2^) = 0.165
*S* = 1.07
5704 reflections
407 parameters
2 restraints
H atoms treated by a mixture of independent and constrained refinement
Δρ_max_ = 0.31 e Å^−3^
Δρ_min_ = −0.24 e Å^−3^

### 

Data collection: *APEX2* (Bruker, 2009 ▶); cell refinement: *SAINT* (Bruker, 2009 ▶); data reduction: *SAINT*; program(s) used to solve structure: *SHELXL97* (Sheldrick, 2008 ▶); program(s) used to refine structure: *SHELXS97* (Sheldrick, 2008 ▶); molecular graphics: *SHELXTL* (Sheldrick, 2008 ▶); software used to prepare material for publication: *SHELXTL*.

## Supplementary Material

Crystal structure: contains datablocks global, I. DOI: 10.1107/S1600536811000201/su2244sup1.cif

Structure factors: contains datablocks I. DOI: 10.1107/S1600536811000201/su2244Isup2.hkl

Additional supplementary materials:  crystallographic information; 3D view; checkCIF report

## Figures and Tables

**Table 1:** Hydrogen-bond geometry (Å, °)

*D*—H⋯*A*	*D*—H	H⋯*A*	*D*⋯*A*	*D*—H⋯*A*
O1—H1*A*⋯O7^i^	0.82	1.86	2.650 (3)	162
O5—H5⋯O4	0.82	1.94	2.621 (3)	140
O5—H5⋯O4^ii^	0.82	2.30	2.997 (3)	143
O5—H5⋯N3	0.82	2.51	2.928 (3)	112
O6—H6⋯O2	0.82	1.91	2.713 (3)	168
O10—H10⋯O9	0.82	1.91	2.596 (3)	140
O10—H10⋯N6	0.82	2.50	2.909 (4)	112
N4—H4⋯O6	0.90 (1)	1.93 (3)	2.640 (3)	134 (3)
N1—H1⋯O1	0.90 (1)	1.84 (2)	2.599 (3)	141 (3)

Symmetry codes: (i) 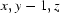; (ii) 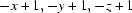.

**Fig. 1.**
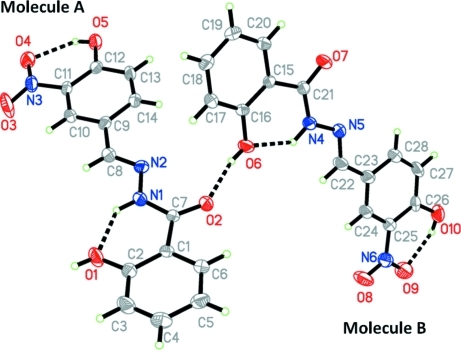
A view of the molecular structure of the title compound, showing displacement ellipsoids at the 30% probability level. Intramolecular O—H···O and N—H···O hydrogen bonds are shown as dashed lines.

## supplementary crystallographic information

### Comment

Hydrazone compounds have received much attention due to their potential applications in biological chemistry (Ajani *et al.*, 2010; Avaji *et al.*, 2009; Fan *et al.*, 2010; Rasras *et al.*, 2010). As a continuous work on the hydrazone compounds, a new hydrazone compound, *N*'-(4-hydroxy-3-nitrobenzylidene)-2-hydroxybenzohydrazide, was prepared and structurally characterized.

The title compound contains two independent molecules (Fig. 1). Each molecule (A abd B) assumes an *E* configuration with respect to the methylidene unit. Intramolecular O—H···O and N—H···O hydrogen bonds are present in the molecules and they are linked by a O-H···O hydrogen bond (Table 1 and Fig. 1). The bond lengths are comparable to those observed in similar hydrazone compounds (Ahmad *et al.*, 2010; Ban, 2010; Ji & Lu, 2010; Shalash *et al.*, 2010). The dihedral angles between the mean planes of the two benzene rings are 4.45 (16)) and 1.7 (2)° for molecules A and B, respectively.

The crystal structure is stabilized by intermolecular O—H···O and N—H···O hydrogen bonds (Table 1).

### Experimental

An ethanol solution (50 ml) of 2-hydroxybenzohydrazide (0.01 mol) and 4-hydroxy-3-nitrobenzaldehyde (0.01 mol) was stirred at room temperature for 30 min to give a yellow solution. Yellow block-shaped single crystals, suitable for X-ray diffraction, were formed by slow evaporation of the solution in air.

### Refinement

The amino H-atoms, H1 and H4, were located from a difference Fourier map and were refined with N—H distance restraints of 0.90 (1) Å. The remaining H atoms were positioned geometrically and refined using the riding-model approximation: C—H = 0.93 Å, and O—H = 0.82 Å, with *U*_iso_(H) = 1.2*U*_eq_(parent C-atoms) and 1.5*U*_eq_(parent O-atom).

### Crystal data

**Table:**

C_14_H_11_N_3_O_5_	*F*(000) = 1248
*M**_r_* = 301.26	*D*_x_ = 1.481 Mg m^−^^3^
Monoclinic, *P*2/*c*	Mo *K*α radiation, λ = 0.71073 Å
*a* = 13.769 (2) Å	Cell parameters from 2499 reflections
*b* = 13.089 (2) Å	θ = 2.5–24.5°
*c* = 19.999 (3) Å	µ = 0.12 mm^−^^1^
β = 131.426 (3)°	*T* = 298 K
*V* = 2702.5 (7) Å^3^	Block, yellow
*Z* = 8	0.23 × 0.21 × 0.20 mm

### Data collection

**Table:**

Bruker SMART APEX CCD area-detector diffractometer	5704 independent reflections
Radiation source: fine-focus sealed tube	3336 reflections with *I* > 2σ(*I*)
graphite	*R*_int_ = 0.063
ω scans	θ_max_ = 27.0°, θ_min_ = 1.6°
Absorption correction: multi-scan (*SADABS*; Bruker, 2009)	*h* = −17→17
*T*_min_ = 0.974, *T*_max_ = 0.977	*k* = −16→16
20705 measured reflections	*l* = −25→25

### Refinement

**Table:**

Refinement on *F*^2^	Primary atom site location: structure-invariant direct methods
Least-squares matrix: full	Secondary atom site location: difference Fourier map
*R*[*F*^2^ > 2σ(*F*^2^)] = 0.074	Hydrogen site location: inferred from neighbouring sites
*wR*(*F*^2^) = 0.165	H atoms treated by a mixture of independent and constrained refinement
*S* = 1.07	*w* = 1/[σ^2^(*F*_o_^2^) + (0.059*P*)^2^ + 0.7268*P*] where *P* = (*F*_o_^2^ + 2*F*_c_^2^)/3
5704 reflections	(Δ/σ)_max_ < 0.001
407 parameters	Δρ_max_ = 0.31 e Å^−^^3^
2 restraints	Δρ_min_ = −0.24 e Å^−^^3^

### Special details

**Table:**

Geometry. All e.s.d.'s (except the e.s.d. in the dihedral angle between two l.s. planes) are estimated using the full covariance matrix. The cell e.s.d.'s are taken into account individually in the estimation of e.s.d.'s in distances, angles and torsion angles; correlations between e.s.d.'s in cell parameters are only used when they are defined by crystal symmetry. An approximate (isotropic) treatment of cell e.s.d.'s is used for estimating e.s.d.'s involving l.s. planes.
Refinement. Refinement of *F*^2^ against ALL reflections. The weighted *R*-factor *wR* and goodness of fit *S* are based on *F*^2^, conventional *R*-factors *R* are based on *F*, with *F* set to zero for negative *F*^2^. The threshold expression of *F*^2^ > σ(*F*^2^) is used only for calculating *R*-factors(gt) *etc*. and is not relevant to the choice of reflections for refinement. *R*-factors based on *F*^2^ are statistically about twice as large as those based on *F*, and *R*- factors based on ALL data will be even larger.

### Fractional atomic coordinates and isotropic or equivalent isotropic displacement parameters (Å^2^)

**Table:**

	*x*	*y*	*z*	*U*_iso_*/*U*_eq_	
O1	0.2000 (3)	0.18864 (15)	0.83343 (17)	0.0670 (7)	
H1A	0.1778	0.1291	0.8178	0.100*	
O2	0.1875 (2)	0.50089 (14)	0.86317 (14)	0.0588 (6)	
O3	0.4530 (4)	0.29706 (19)	0.5827 (2)	0.1199 (13)	
O4	0.4754 (2)	0.42998 (17)	0.53343 (16)	0.0676 (7)	
O5	0.4360 (2)	0.61199 (15)	0.56619 (16)	0.0591 (6)	
H5	0.4648	0.5741	0.5502	0.089*	
O6	0.2115 (2)	0.69519 (14)	0.82854 (13)	0.0543 (6)	
H6	0.2037	0.6340	0.8329	0.082*	
O7	0.1398 (2)	1.00436 (14)	0.76080 (14)	0.0621 (7)	
O8	0.7151 (3)	0.85882 (19)	1.33786 (16)	0.0998 (10)	
O9	0.8001 (3)	0.99999 (19)	1.40763 (16)	0.0896 (9)	
O10	0.7080 (2)	1.17289 (16)	1.32351 (15)	0.0645 (6)	
H10	0.7641	1.1385	1.3674	0.097*	
N1	0.2146 (3)	0.37642 (17)	0.79853 (17)	0.0477 (6)	
N2	0.2490 (2)	0.44197 (17)	0.76341 (15)	0.0450 (6)	
N3	0.4419 (3)	0.3884 (2)	0.56981 (18)	0.0582 (7)	
N4	0.2472 (3)	0.89030 (17)	0.87275 (16)	0.0480 (6)	
N5	0.3115 (2)	0.96498 (17)	0.93740 (17)	0.0465 (6)	
N6	0.7213 (3)	0.9512 (2)	1.33793 (19)	0.0682 (8)	
C1	0.1415 (3)	0.3304 (2)	0.87495 (17)	0.0387 (7)	
C2	0.1469 (3)	0.2249 (2)	0.86681 (19)	0.0457 (7)	
C3	0.1009 (3)	0.1584 (2)	0.8941 (2)	0.0602 (9)	
H3	0.1042	0.0883	0.8881	0.072*	
C4	0.0508 (3)	0.1945 (3)	0.9297 (2)	0.0618 (9)	
H4A	0.0202	0.1488	0.9477	0.074*	
C5	0.0451 (3)	0.2982 (3)	0.9393 (2)	0.0609 (9)	
H5A	0.0113	0.3228	0.9639	0.073*	
C6	0.0900 (3)	0.3642 (2)	0.91194 (19)	0.0509 (8)	
H6A	0.0860	0.4341	0.9183	0.061*	
C7	0.1842 (3)	0.4105 (2)	0.84622 (17)	0.0398 (7)	
C8	0.2731 (3)	0.3978 (2)	0.7192 (2)	0.0498 (8)	
H8	0.2666	0.3270	0.7141	0.060*	
C9	0.3104 (3)	0.4536 (2)	0.67645 (18)	0.0418 (7)	
C10	0.3520 (3)	0.3994 (2)	0.64042 (19)	0.0468 (8)	
H10A	0.3514	0.3284	0.6412	0.056*	
C11	0.3948 (3)	0.4498 (2)	0.60285 (18)	0.0432 (7)	
C12	0.3946 (3)	0.5558 (2)	0.59936 (18)	0.0425 (7)	
C13	0.3491 (3)	0.6095 (2)	0.6339 (2)	0.0488 (8)	
H13	0.3462	0.6805	0.6311	0.059*	
C14	0.3088 (3)	0.5604 (2)	0.67171 (19)	0.0469 (8)	
H14	0.2798	0.5984	0.6948	0.056*	
C15	0.0970 (3)	0.8290 (2)	0.72103 (18)	0.0370 (7)	
C16	0.1181 (3)	0.7249 (2)	0.74221 (19)	0.0391 (7)	
C17	0.0450 (3)	0.6528 (2)	0.6754 (2)	0.0500 (8)	
H17	0.0587	0.5837	0.6899	0.060*	
C18	−0.0476 (3)	0.6822 (2)	0.5879 (2)	0.0571 (9)	
H18	−0.0967	0.6329	0.5436	0.069*	
C19	−0.0682 (3)	0.7839 (3)	0.5651 (2)	0.0579 (9)	
H19	−0.1298	0.8036	0.5057	0.070*	
C20	0.0033 (3)	0.8557 (2)	0.6311 (2)	0.0470 (8)	
H20	−0.0110	0.9245	0.6155	0.056*	
C21	0.1630 (3)	0.9152 (2)	0.7860 (2)	0.0426 (7)	
C22	0.3914 (3)	0.9314 (2)	1.0165 (2)	0.0625 (10)	
H22	0.3997	0.8609	1.0246	0.075*	
C23	0.4704 (3)	0.9952 (2)	1.0952 (2)	0.0507 (8)	
C24	0.5549 (3)	0.9492 (2)	1.1772 (2)	0.0553 (9)	
H24	0.5581	0.8782	1.1807	0.066*	
C25	0.6354 (3)	1.0058 (2)	1.2550 (2)	0.0477 (8)	
C26	0.6330 (3)	1.1123 (2)	1.2519 (2)	0.0474 (8)	
C27	0.5487 (3)	1.1584 (2)	1.1691 (2)	0.0546 (9)	
H27	0.5465	1.2294	1.1655	0.066*	
C28	0.4687 (3)	1.1026 (2)	1.0926 (2)	0.0535 (8)	
H28	0.4123	1.1360	1.0379	0.064*	
H4	0.270 (3)	0.8247 (11)	0.891 (2)	0.080*	
H1	0.212 (3)	0.3085 (9)	0.791 (2)	0.080*	

### Atomic displacement parameters (Å^2^)

**Table:**

	*U*^11^	*U*^22^	*U*^33^	*U*^12^	*U*^13^	*U*^23^
O1	0.106 (2)	0.0290 (11)	0.1044 (19)	−0.0067 (12)	0.0862 (18)	−0.0046 (12)
O2	0.1030 (19)	0.0292 (11)	0.0670 (15)	0.0012 (11)	0.0659 (15)	0.0023 (10)
O3	0.236 (4)	0.0329 (15)	0.207 (4)	−0.0015 (18)	0.196 (3)	−0.0109 (17)
O4	0.102 (2)	0.0600 (15)	0.0793 (17)	0.0028 (13)	0.0762 (17)	−0.0017 (12)
O5	0.0926 (18)	0.0406 (12)	0.0772 (16)	−0.0044 (12)	0.0703 (15)	−0.0040 (11)
O6	0.0733 (15)	0.0324 (11)	0.0437 (12)	−0.0010 (10)	0.0329 (12)	0.0067 (9)
O7	0.0910 (18)	0.0302 (12)	0.0582 (14)	0.0079 (11)	0.0464 (14)	0.0088 (10)
O8	0.144 (3)	0.0431 (15)	0.0596 (16)	0.0069 (15)	0.0448 (18)	0.0106 (12)
O9	0.108 (2)	0.0700 (18)	0.0492 (15)	0.0010 (16)	0.0340 (16)	−0.0077 (14)
O10	0.0732 (17)	0.0485 (13)	0.0689 (16)	−0.0015 (11)	0.0457 (14)	−0.0152 (12)
N1	0.0712 (18)	0.0307 (13)	0.0543 (16)	−0.0025 (13)	0.0470 (15)	0.0010 (12)
N2	0.0614 (17)	0.0371 (14)	0.0429 (14)	−0.0034 (12)	0.0371 (14)	0.0033 (11)
N3	0.088 (2)	0.0385 (16)	0.0702 (19)	−0.0048 (14)	0.0617 (19)	−0.0090 (14)
N4	0.0687 (18)	0.0288 (13)	0.0442 (15)	−0.0018 (13)	0.0363 (15)	0.0000 (12)
N5	0.0598 (17)	0.0347 (13)	0.0506 (16)	0.0011 (12)	0.0389 (15)	0.0003 (12)
N6	0.092 (2)	0.0496 (18)	0.0515 (19)	0.0032 (17)	0.0423 (19)	−0.0013 (15)
C1	0.0458 (18)	0.0343 (15)	0.0319 (15)	0.0022 (13)	0.0240 (15)	0.0047 (12)
C2	0.055 (2)	0.0394 (17)	0.0470 (18)	−0.0043 (14)	0.0354 (17)	−0.0009 (14)
C3	0.071 (2)	0.0397 (18)	0.074 (2)	−0.0056 (16)	0.050 (2)	0.0032 (16)
C4	0.065 (2)	0.060 (2)	0.067 (2)	−0.0036 (18)	0.046 (2)	0.0138 (18)
C5	0.076 (3)	0.069 (2)	0.058 (2)	0.0092 (19)	0.053 (2)	0.0136 (18)
C6	0.069 (2)	0.0422 (17)	0.0472 (18)	0.0098 (15)	0.0409 (18)	0.0092 (14)
C7	0.0517 (19)	0.0307 (15)	0.0330 (15)	0.0028 (13)	0.0264 (15)	0.0033 (12)
C8	0.065 (2)	0.0366 (16)	0.0512 (19)	−0.0047 (15)	0.0402 (19)	−0.0023 (14)
C9	0.0488 (19)	0.0411 (16)	0.0340 (16)	−0.0051 (14)	0.0268 (15)	−0.0053 (13)
C10	0.064 (2)	0.0286 (15)	0.0454 (18)	−0.0038 (14)	0.0348 (17)	−0.0031 (13)
C11	0.059 (2)	0.0355 (16)	0.0425 (17)	−0.0022 (14)	0.0365 (17)	−0.0071 (13)
C12	0.054 (2)	0.0386 (16)	0.0389 (17)	−0.0060 (14)	0.0324 (16)	−0.0035 (13)
C13	0.072 (2)	0.0296 (15)	0.058 (2)	−0.0021 (15)	0.0486 (19)	−0.0044 (14)
C14	0.065 (2)	0.0380 (17)	0.0490 (18)	0.0005 (14)	0.0423 (18)	−0.0032 (14)
C15	0.0509 (18)	0.0341 (15)	0.0427 (17)	0.0035 (13)	0.0381 (16)	0.0051 (12)
C16	0.0521 (19)	0.0352 (15)	0.0412 (17)	0.0038 (14)	0.0355 (16)	0.0072 (13)
C17	0.068 (2)	0.0314 (15)	0.052 (2)	0.0003 (15)	0.0404 (19)	0.0002 (14)
C18	0.071 (2)	0.052 (2)	0.0433 (19)	−0.0020 (17)	0.0358 (19)	−0.0067 (16)
C19	0.067 (2)	0.063 (2)	0.0369 (18)	0.0123 (18)	0.0312 (18)	0.0081 (16)
C20	0.061 (2)	0.0405 (17)	0.0484 (18)	0.0102 (15)	0.0402 (18)	0.0099 (15)
C21	0.056 (2)	0.0362 (16)	0.0511 (19)	0.0041 (14)	0.0418 (18)	0.0041 (14)
C22	0.089 (3)	0.0291 (16)	0.050 (2)	0.0015 (16)	0.038 (2)	0.0028 (15)
C23	0.063 (2)	0.0377 (17)	0.0480 (19)	−0.0021 (15)	0.0353 (18)	0.0006 (15)
C24	0.078 (2)	0.0312 (16)	0.053 (2)	0.0012 (16)	0.0414 (19)	0.0007 (15)
C25	0.055 (2)	0.0412 (17)	0.0488 (19)	0.0033 (15)	0.0349 (18)	0.0023 (15)
C26	0.054 (2)	0.0385 (17)	0.062 (2)	0.0003 (15)	0.0438 (19)	−0.0102 (16)
C27	0.066 (2)	0.0302 (16)	0.074 (2)	0.0070 (15)	0.049 (2)	0.0027 (16)
C28	0.066 (2)	0.0382 (17)	0.057 (2)	0.0048 (16)	0.0414 (19)	0.0063 (16)

### Geometric parameters (Å, °)

**Table:**

O1—C2	1.361 (3)	C6—H6A	0.9300
O1—H1A	0.8200	C8—C9	1.454 (4)
O2—C7	1.224 (3)	C8—H8	0.9300
O3—N3	1.211 (3)	C9—C10	1.377 (4)
O4—N3	1.217 (3)	C9—C14	1.400 (4)
O5—C12	1.344 (3)	C10—C11	1.391 (4)
O5—H5	0.8200	C10—H10A	0.9300
O6—C16	1.359 (3)	C11—C12	1.389 (4)
O6—H6	0.8200	C12—C13	1.391 (4)
O7—C21	1.227 (3)	C13—C14	1.358 (4)
O8—N6	1.213 (3)	C13—H13	0.9300
O9—N6	1.235 (3)	C14—H14	0.9300
O10—C26	1.337 (3)	C15—C20	1.397 (4)
O10—H10	0.8200	C15—C16	1.399 (4)
N1—C7	1.346 (3)	C15—C21	1.491 (4)
N1—N2	1.377 (3)	C16—C17	1.381 (4)
N1—H1	0.898 (10)	C17—C18	1.373 (4)
N2—C8	1.269 (3)	C17—H17	0.9300
N3—C11	1.439 (4)	C18—C19	1.374 (4)
N4—C21	1.342 (4)	C18—H18	0.9300
N4—N5	1.377 (3)	C19—C20	1.369 (4)
N4—H4	0.903 (10)	C19—H19	0.9300
N5—C22	1.266 (4)	C20—H20	0.9300
N6—C25	1.438 (4)	C22—C23	1.446 (4)
C1—C6	1.393 (4)	C22—H22	0.9300
C1—C2	1.398 (4)	C23—C24	1.371 (4)
C1—C7	1.490 (4)	C23—C28	1.407 (4)
C2—C3	1.385 (4)	C24—C25	1.384 (4)
C3—C4	1.361 (4)	C24—H24	0.9300
C3—H3	0.9300	C25—C26	1.394 (4)
C4—C5	1.381 (5)	C26—C27	1.383 (4)
C4—H4A	0.9300	C27—C28	1.364 (4)
C5—C6	1.370 (4)	C27—H27	0.9300
C5—H5A	0.9300	C28—H28	0.9300
			
C2—O1—H1A	109.5	O5—C12—C11	125.7 (3)
C12—O5—H5	109.5	O5—C12—C13	116.5 (3)
C16—O6—H6	109.5	C11—C12—C13	117.8 (3)
C26—O10—H10	109.5	C14—C13—C12	121.4 (3)
C7—N1—N2	121.9 (2)	C14—C13—H13	119.3
C7—N1—H1	117 (2)	C12—C13—H13	119.3
N2—N1—H1	121 (2)	C13—C14—C9	121.1 (3)
C8—N2—N1	114.1 (2)	C13—C14—H14	119.5
O3—N3—O4	121.6 (3)	C9—C14—H14	119.5
O3—N3—C11	119.1 (3)	C20—C15—C16	117.6 (3)
O4—N3—C11	119.2 (3)	C20—C15—C21	116.3 (2)
C21—N4—N5	120.7 (2)	C16—C15—C21	126.0 (3)
C21—N4—H4	121 (2)	O6—C16—C17	120.3 (2)
N5—N4—H4	118 (2)	O6—C16—C15	119.7 (3)
C22—N5—N4	114.5 (2)	C17—C16—C15	120.0 (3)
O8—N6—O9	121.7 (3)	C18—C17—C16	120.6 (3)
O8—N6—C25	119.4 (3)	C18—C17—H17	119.7
O9—N6—C25	118.9 (3)	C16—C17—H17	119.7
C6—C1—C2	117.4 (3)	C17—C18—C19	120.5 (3)
C6—C1—C7	116.8 (2)	C17—C18—H18	119.7
C2—C1—C7	125.8 (3)	C19—C18—H18	119.7
O1—C2—C3	120.6 (3)	C20—C19—C18	119.1 (3)
O1—C2—C1	119.3 (3)	C20—C19—H19	120.4
C3—C2—C1	120.1 (3)	C18—C19—H19	120.4
C4—C3—C2	120.7 (3)	C19—C20—C15	122.1 (3)
C4—C3—H3	119.7	C19—C20—H20	119.0
C2—C3—H3	119.7	C15—C20—H20	119.0
C3—C4—C5	120.7 (3)	O7—C21—N4	121.9 (3)
C3—C4—H4A	119.7	O7—C21—C15	121.3 (3)
C5—C4—H4A	119.7	N4—C21—C15	116.7 (2)
C6—C5—C4	118.8 (3)	N5—C22—C23	124.4 (3)
C6—C5—H5A	120.6	N5—C22—H22	117.8
C4—C5—H5A	120.6	C23—C22—H22	117.8
C5—C6—C1	122.3 (3)	C24—C23—C28	117.6 (3)
C5—C6—H6A	118.8	C24—C23—C22	118.7 (3)
C1—C6—H6A	118.8	C28—C23—C22	123.6 (3)
O2—C7—N1	123.0 (3)	C23—C24—C25	121.5 (3)
O2—C7—C1	121.7 (3)	C23—C24—H24	119.2
N1—C7—C1	115.3 (2)	C25—C24—H24	119.2
N2—C8—C9	122.5 (3)	C24—C25—C26	120.6 (3)
N2—C8—H8	118.8	C24—C25—N6	117.8 (3)
C9—C8—H8	118.8	C26—C25—N6	121.6 (3)
C10—C9—C14	118.2 (3)	O10—C26—C27	117.7 (3)
C10—C9—C8	118.7 (3)	O10—C26—C25	124.6 (3)
C14—C9—C8	123.1 (3)	C27—C26—C25	117.7 (3)
C9—C10—C11	120.7 (3)	C28—C27—C26	121.7 (3)
C9—C10—H10A	119.7	C28—C27—H27	119.2
C11—C10—H10A	119.7	C26—C27—H27	119.2
C12—C11—C10	120.9 (3)	C27—C28—C23	120.8 (3)
C12—C11—N3	121.5 (3)	C27—C28—H28	119.6
C10—C11—N3	117.7 (3)	C23—C28—H28	119.6

### Hydrogen-bond geometry (Å, °)

**Table:**

*D*—H···*A*	*D*—H	H···*A*	*D*···*A*	*D*—H···*A*
O1—H1A···O7^i^	0.82	1.86	2.650 (3)	162
O5—H5···O4	0.82	1.94	2.621 (3)	140
O5—H5···O4^ii^	0.82	2.30	2.997 (3)	143
O5—H5···N3	0.82	2.51	2.928 (3)	112
O6—H6···O2	0.82	1.91	2.713 (3)	168
O10—H10···O9	0.82	1.91	2.596 (3)	140
O10—H10···N6	0.82	2.50	2.909 (4)	112
N4—H4···O6	0.90 (1)	1.93 (3)	2.640 (3)	134 (3)
N1—H1···O1	0.90 (1)	1.84 (2)	2.599 (3)	141 (3)

Symmetry codes: (i) *x*, *y*−1, *z*; (ii) −*x*+1, −*y*+1, −*z*+1.
